# Probing the Anti-Cancer Potency of Sulfated Galactans on Cholangiocarcinoma Cells Using Synchrotron FTIR Microspectroscopy, Molecular Docking, and In Vitro Studies

**DOI:** 10.3390/md19050258

**Published:** 2021-04-30

**Authors:** Boonyakorn Boonsri, Kiattawee Choowongkomon, Buabarn Kuaprasert, Thanvarin Thitiphatphuvanon, Kittiya Supradit, Apinya Sayinta, Jinchutha Duangdara, Tawut Rudtanatip, Kanokpan Wongprasert

**Affiliations:** 1 Department of Anatomy, Faculty of Science, Mahidol University, Bangkok 10400, Thailand; nantavadee.bon@student.mahidol.ac.th (B.B.); kittiya.sup@student.mahidol.ac.th (K.S.); apinya.say@student.mahidol.ac.th (A.S.); jinchutha.dua@student.mahidol.ac.th (J.D.); 2 Department of Biochemistry, Faculty of Science, Kasetsart University, Bangkok 10900, Thailand; fsciktc@ku.ac.th; 3 Research and Facility Division, Synchrotron Light Research Institute (Public Organization), Nakhorn Ratchasima 30000, Thailand; buabarn@slri.or.th; 4 Department of Anatomy, Faculty of Medicine, Siam University, Bangkok 10160, Thailand; thannicha.sae@siam.edu; 5 Department of Anatomy, Faculty of Medicine, Khon Kean University, Khon Kean 40002, Thailand; tawut@kku.ac.th

**Keywords:** red alga *Gracilaria fisheri*, sulfated galactans, synchrotron-FTIR-MS, molecular docking, epidermal growth factor receptor, anti-cancer

## Abstract

Sulfated galactans (SG) isolated from red alga *Gracilaria fisheri* have been reported to inhibit the growth of cholangiocarcinoma (CCA) cells, which was similar to the epidermal growth factor receptor (EGFR)-targeted drug, cetuximab. Herein, we studied the anti-cancer potency of SG compared to cetuximab. Biological studies demonstrated SG and cetuximab had similar inhibition mechanisms in CCA cells by down-regulating EGFR/ERK pathway, and the combined treatment induced a greater inhibition effect. The molecular docking study revealed that SG binds to the dimerization domain of EGFR, and this was confirmed by dimerization assay, which showed that SG inhibited ligand-induced EGFR dimer formation. Synchrotron FTIR microspectroscopy was employed to examine alterations in cellular macromolecules after drug treatment. The SR-FTIR-MS elicited similar spectral signatures of SG and cetuximab, pointing towards the bands of RNA/DNA, lipids, and amide I vibrations, which were inconsistent with the changes of signaling proteins in CCA cells after drug treatment. Thus, this study demonstrates the underlined anti-cancer mechanism of SG by interfering with EGFR dimerization. In addition, we reveal that FTIR signature spectra offer a useful tool for screening anti-cancer drugs’ effect.

## 1. Introduction

Cholangiocarcinoma (CCA) is a malignant cancer of the bile duct epithelium. The largest incidence of CCA has been reported in the northeast part of Thailand (135.4 per 100,000) [1], where development of CCA has been shown to correlate with liver fluke (*Opistorchis viverrini*) infection. To date, there are no efficient tools for early detection and no effective treatment strategies for CCA patients [2,3].

The epidermal growth factor receptor (EGFR) is a tyrosine kinase receptor and is overexpressed in many tumor cell types, including CCA. As such, EGFR is one of the most common and promising target proteins in anti-cancer therapy. The epidermal growth factor (EGF) is a ligand that activates EGFR by binding to the EGFR extracellular domains I and III and promotes a conformational transition from a closed, self-inhibited tethered form locked by the molecular interaction between domain II and IV to an open untethered form. This extracellular domain rearrangement allows domains II and IV to bind to the corresponding domains of the adjacent receptor facilitating homo- or hetero-dimerization, auto-phosphorylation, and the activation of signaling pathways that regulate cancer cell proliferation and migration [4]. Cetuximab is a chimeric monoclonal antibody targeted to EGFR by preferential binding to domain III of the extracellular domain, which prevents ligand binding [5,6]. Cetuximab treatment provides some benefit to CCA patients, but resistance to the drug has become a challenging problem. Several reasons have been proposed to explain the therapeutic failure of EGFR-targeted therapies, and new treatment options or drug adjuvants for CCA are currently under investigation.

Sulfated galactans (SG) is a sulfated polysaccharide isolated from the edible red alga *Gracilaria fisheri* [7]. It has shown various biological activities, including anti-viral [7], anti-oxidant [8], immune stimulant [9], and anti-cancer [10,11]. Recently, we have demonstrated the anti-proliferation effect of SG against CCA cells, which is similar to cetuximab [10,11]. We hypothesized that SG potentially mediates an anti-tumor effect similar to cetuximab via interacting with the EGFR extracellular domain.

Synchrotron radiation-based FTIR microspectroscopy (SR-FTIR-MS) is an excellent methodology that combines synchrotron radiation and microspectroscopy and has been applied in modern biological research for studying molecular reactions in cells. The assessment of cell functionality analysis was analyzed based on the vibrational transition of chemical bonding that reacted with an infrared in individual living cells or tissue sections [12,13,14]. The FTIR spectra for the study of the biological material comprise the range between 3000–800 cm^−1^. Wavenumbers 2800–3000 cm^−1^ represent changes associated with lipid structure, the region of 1500–1700 cm^−1^ is correlated with the secondary structure of proteins, and the fingerprint region (800–1450 cm^−1^) is related to the changes of nucleic acid and carbohydrate structure [15,16]. In cancer research, SR-FTIR-MS has been used as a non-labeled tool for preclinical screening of anti-cancer agents and discriminating chemo-sensitive and chemo-resistant cells in different cancers [13,17]. Moreover, recent studies have reported the FTIR signature spectra that correlated with apoptotic CCA cells [18] and for monitoring of CCA progression in the hamster model [19].

In this study, we used multidisciplinary tools to evaluate the anti-cancer potency and underlined molecular mechanism of SG, including in vitro biological anti-migration assay, molecular docking analysis for predicting the binding site of SG on EGFR, and SR-FTIR-MS for investigating the biochemical composition changes after drug treatment.

## 2. Results

### 2.1. SG and Cetuximab Inhibited CCA Cell Viability

**Figure 1:**
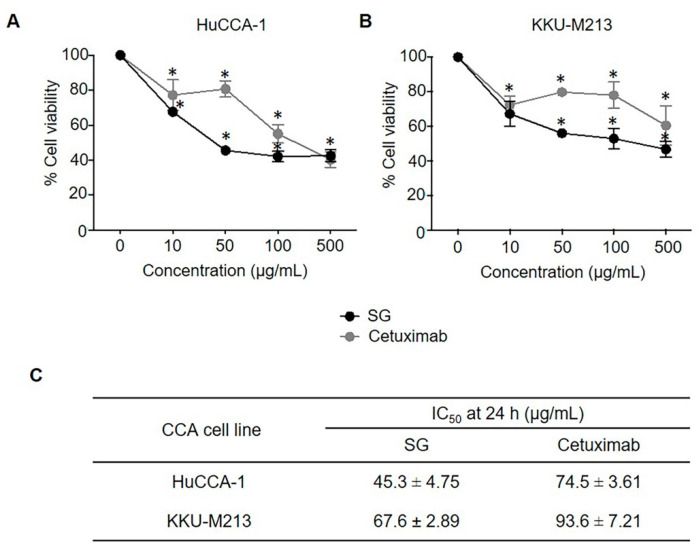
The cytotoxic effect of SG and cetuximab on CCA cell lines. CCA cells were treated with the indicated concentrations of SG and cetuximab (10, 50, 100, and 500 μg/mL) in a medium containing 1% FBS. Cell viability was determined using an MTT assay. SG and cetuximab decreased (**A**) HuCCA-1 and (**B**) KKU-M213 cell viability. (**C**) Mean IC50 values (µg/mL) for SG and cetuximab tested against CCA cell lines at 24 h. Data are representative of mean ± SEM from three independent experiments. * p < 0.05 compared with the control group.

The cytotoxic effect of SG on CCA cells was investigated and compared to cetuximab. HuCCA-1 and KKU-M213 cells were treated with SG or cetuximab for 24 h and analyzed by the MTT assay. We found that both SG and cetuximab produced a dose-dependent cytotoxic effect on the two cell lines tested; HuCCA-1 (Figure 1A), KKU-M213 (Figure 1B). The IC_50_ values obtained from each independent treatment for the two cell lines are shown in Figure 1C. IC_50_ values for SG for the cell lines ranged from about 45 to 68 μg/mL; for cetuximab, they ranged from about 75 to 94 μg/mL.

### 2.2. SG, Cetuximab, and Combination of SG and Cetuximab Inhibit CCA Cell Migration

**Figure 2:**
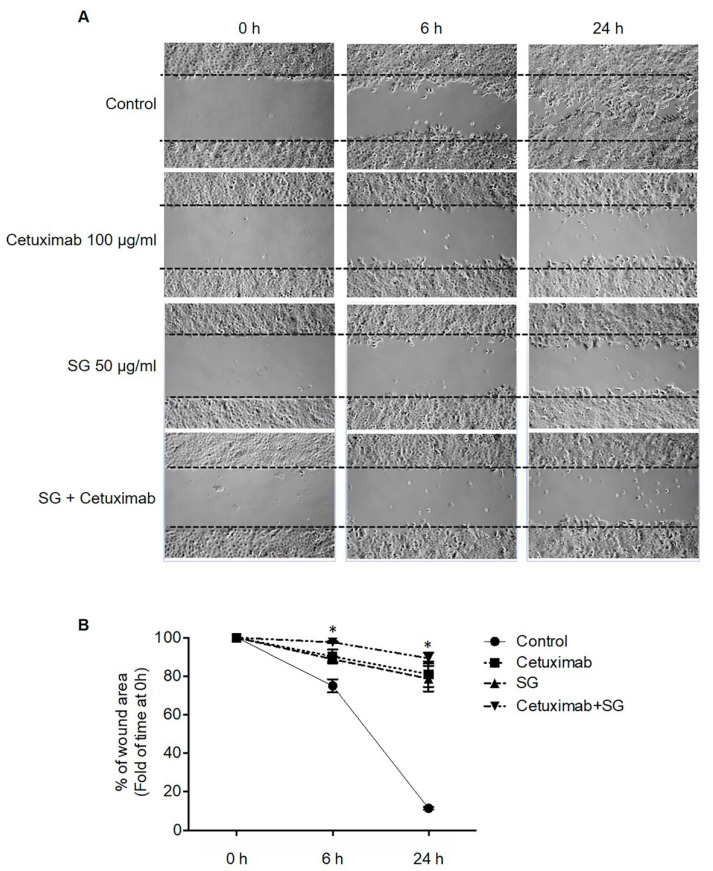
The effect of cetuximab, SG, and a combination of cetuximab and SG on HuCCA-1 cell migration by scratch wound-healing assay. Cells were scratch wounded and treated with cetuximab (100 μg/mL), SG (50 μg/mL), or a combination of cetuximab and SG. Photographs were recorded at 0, 6, and 24 h after scratching. (**A**) Phase-contrast micrographs showing the scratch wound area in different treatment groups compared with control. (**B**) The percent of wound area (fold of time at 0 h) in each time point. Data are representative of mean ± SEM from three independent experiments. * p < 0.05 compared with the control group at each time-point.

We investigated the effect of SG compared to cetuximab, and the efficacy of their combination, on CCA cell migration. The HuCCA-1 cell lines underwent the scratch wounding, followed by treatment with cetuximab 100 μg/mL or SG 50 μg/mL, or SG in combination with cetuximab. The phase-contrast micrographs revealed that, after 24 h, all groups, including SG treated, cetuximab treated and SG in combination with cetuximab treated, showed retarded migration of HuCCA-1 compared with control cells. However, the rates were different (Figure 2A). The percentage of wound area at 6 and 24 h for control cells was 75.0 ± 2.44% and 11.4 ± 0.43%, respectively. For cells treated with cetuximab, the percent of wound area at 6 h and 24 h was 90.3 ± 2.53% and 81.1 ± 4.72%, respectively. For SG-treated cells, the percent of wound area at 6 h and 24 h was 88.8 ± 1.43% and 78.8 ± 6.71%, respectively. Cells treated with SG in combination with cetuximab showed that the percent of wound area at 6 h and 24 h was 97.7 ± 0.38% and 89.4 ± 1.94%, respectively (Figure 2B).

### 2.3. SG, Cetuximab, and Combination of SG and Cetuximab Suppressed HuCCA-1 Cell Migration by Down-Regulating Signaling Molecules in EGFR-ERK Pathway

**Figure 3:**
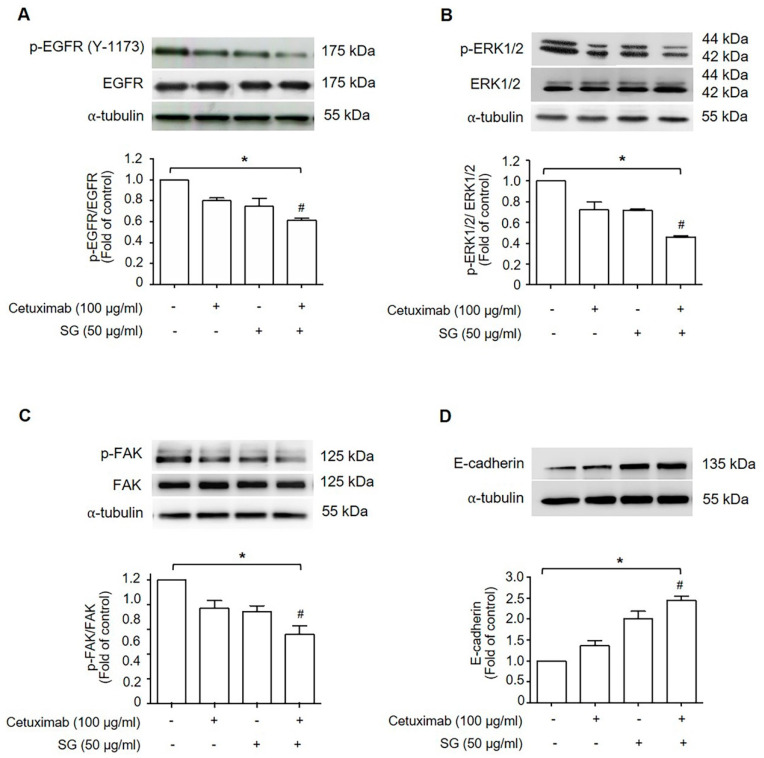
The effect of cetuximab, SG, and a combination of cetuximab and SG on EGFR downstream signaling in HuCCA-1 cells. Cells were treated with 100 μg/mL cetuximab, 50 μg/mL SG, a combination of cetuximab and SG, or left untreated. The phosphorylation and protein levels of (**A**) EGFR, (**B**) ERK1/2, (**C**) FAK, and the protein level of (**D**) E-cadherin were determined by Western blotting analysis. α-tubulin was used as the loading control. Proteins were quantified by densitometric analysis and normalized by α-tubulin. Values are expressed as fold of control (mean ± SEM) from three independent experiments. * *p* < 0.05 compared with the control group and # *p* < 0.05 compared with the cetuximab-treated group.

The EGFR-ERK signaling pathway has been shown to regulate cancer cell migration. We, therefore, investigated the expression of proteins associated with the EGFR-ERK signaling pathway and key regulators of cell migration by Western blot analysis. The results demonstrated that HuCCA-1 cells treated with either cetuximab or SG alone showed a reduction in p-EGFR/EGFR (Figure 3A), p-ERK1/2/ERK1/2 (Figure 3B), and p-FAK/FAK (Figure 3C) but increased the expression of E-cadherin (Figure 3D). Treatment with cetuximab combined with SG produced a greater reduction in p-EGFR/EGFR, p-ERK1/2/ERK1/2, and p-FAK/FAK and increased the expression of E-cadherin, compared to cetuximab treatment alone.

### 2.4. Molecular Docking of SG with the Extracellular Domain of EGFR

**Figure 4:**
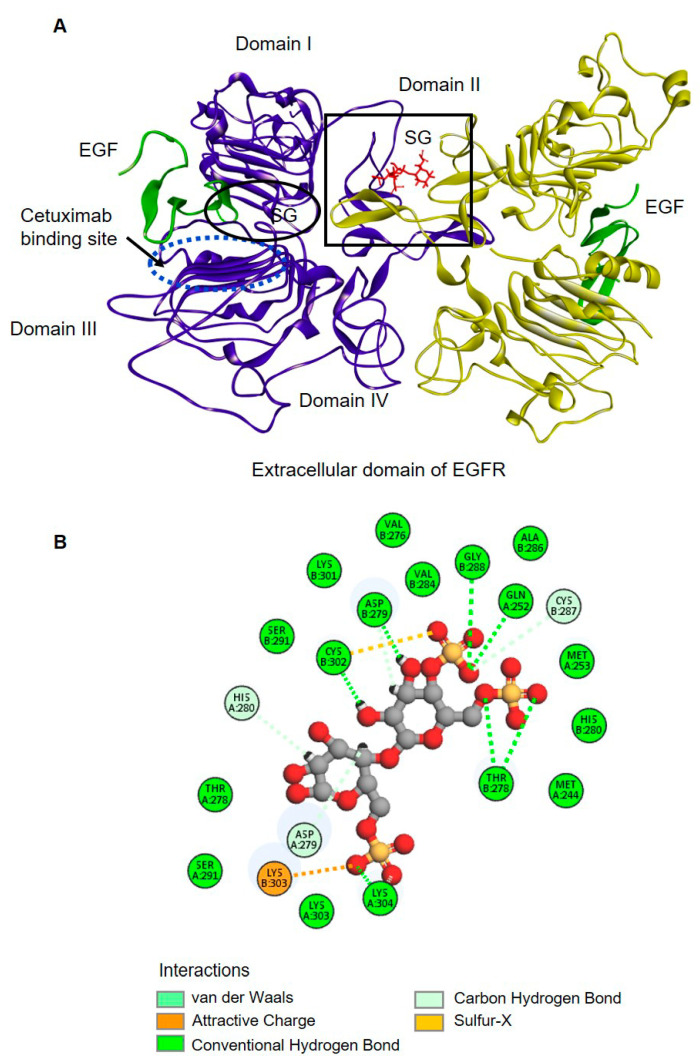
3D docking model of the SG/extracellular domain of the EGFR complex. (**A**) A ribbon representation showing the model of EGFR-EGF-SG complex; purple and yellow represent the dimerization of the extracellular domain of EGFR; green ribbons represent EGF ligands; red color indicates SG. The binding site of SG in the dimer interface is presented in the boundary. (**B**) An interaction of SG with the EGFR domain II (dimerization domain) in the boundary was represented in a 2D interaction plot. The important interactions were highlighted, including conventional hydrogen bond (green dot line), carbon-hydrogen bond (light green dot line), van der Waals (green) and sulfur interaction (orange).

The molecular details of SG binding to the extracellular domain of EGFR can be “virtually” obtained by using a molecular docking technique, GOLD docking software, to identify the binding interface of the extracellular domain of EGFR (PDB: 1MOX) with SG (Figure 4A). The binding of EGF (green) to domain I (site 1) and III (site 2 and 3) within the extracellular region stabilizes an extended conformation and exposes a dimerization arm in domain II which interacts with another EGFR monomer to form an EGFR dimer. A ribbon representation shows the model of the EGFR-EGF-SG complex: SG (red) bound EGFR (purple and yellow) in the extended conformation in domain II of the EGFR dimerization arm (the square boundary).

In domain II at the EGFR dimer interface (Figure 4A; square), the SG molecule provides interactions with EGFR via conventional hydrogen bonds, carbon-hydrogen bonds, a van der Waals bond, and a charge contact (Figure 4B). These molecular dynamics provide an informed view of the interaction of SG with EGFR in domain II of the EGF dimerization arm of HuCCA-1 cells.

### 2.5. SG Inhibited Ligand-Induced EGFR Dimerization

**Figure 5:**
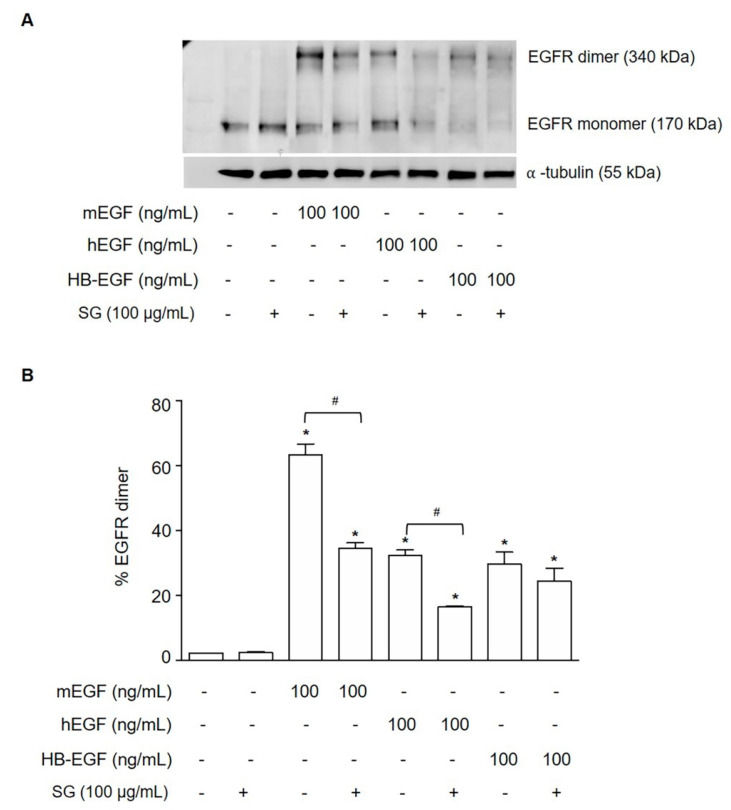
SG inhibited ligand-induced EGFR dimerization. (**A**) Western blot analysis of EGFR monomer and dimer, and α-tubulin in HuCCA-1 cells preincubated with SG for 15 min at a final concentration of 100 μg/mL, and added mEGF, hEGF, and HB-EGF (100 ng/mL) followed by the addition of BS^3^ (3 mM) crosslinker for 20 min on ice. (**B**) Quantitative results were calculated for the percentage of EGFR dimer in each sample. The immunoreactive bands for EGFR monomer and dimer proteins were quantified by densitometric analysis and normalized by α-tubulin. Data are representative of mean ± SEM from three independent experiments. * p < 0.05 compared with cells without treatment and *^#^ p <* 0.05 compared with the respective ligand treatment.

We validated the molecular docking result using an EGFR dimerization cross-linking assay; if SG binds to the dimerization arm of EGFR, the dimer formation of EGFR would be inhibited. HuCCA-1 cells treated with EGFR ligand only were compared with cells pretreated with SG and followed with EGFR ligand. The ligands included mEGF, hEGF, and HB-EGF. The results showed that ligand treatment only markedly increased EGFR dimer, while SG with ligand treatment decreased the percentage of EGFR dimer formation (Figure 5A,B). This suggests that SG is able to inhibit EGFR dimerization.

### 2.6. Synchrotron-FTIR Spectral Signature of HuCCA-1 Cells after Treatment with SG and Cetuximab

**Figure 6:**
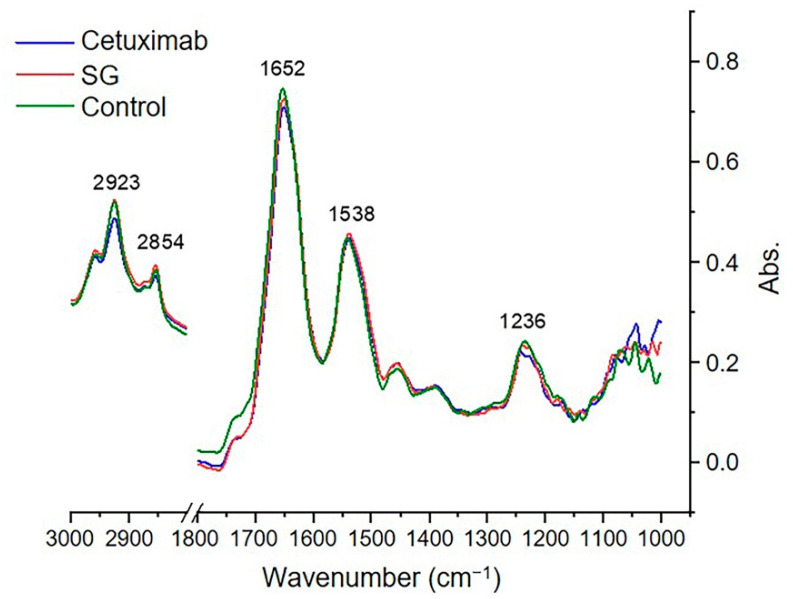
Mean FTIR spectral signature of HuCCA-1 treated with SG, cetuximab, and untreated (control) cells in the wavelength range of 3000–1000 cm^−1^.

SR-FTIR-MS at the single-cell level was used to assess the change in biochemical composition of HuCCA-1 cells treated with SG and cetuximab. Cells were either untreated or treated with 50 µg/mL SG and 100 µg/mL cetuximab for 24 h. Spectra from the SR-FTIR-MS analysis were selected from the untreated and treated cell line individually for further analysis. For overall spectral comparison, the SR-FTIR spectra of each treatment group were taken over the spectral ranges of 3000 to 1000 cm^−1^. They were averaged, normalized and overlaid (Figure 6). The spectral peak that occurred at 2960 and 2923 cm^−1^ corresponded to -CH_3_ and -CH_2_- asymmetric stretching, whereas the spectral peak at 2873 and 2852 cm^−1^ represented -CH_3_ and -CH_2_- symmetric stretching. Alpha helix of amide I was represented in 1656 cm^−1^, and the beta sheet of amide I occurred at 1627 cm^−1^. The spectral peak at 1236 corresponded to an asymmetric stretching of PO^2−^: phospholipids and nucleic acids. Details of the main spectral peaks are presented in Table 1.

### 2.7. PCA Segregation in HuCCA-1 Cells after Treatment with SG and Cetuximab

**Figure 7:**
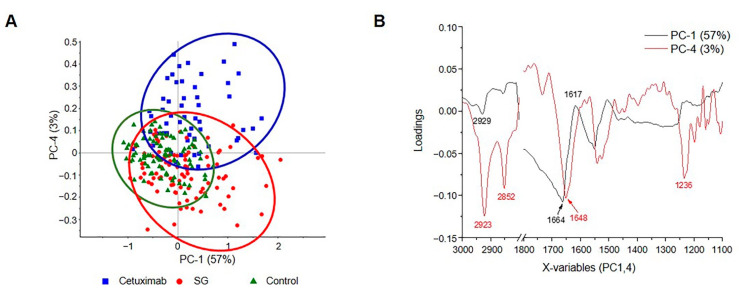
(**A**) Two dimensional PCA analysis of HuCCA-1 untreated cells and HuCCA-1 cells treated with SG and cetuximab using a spectral range of 3000–2819 and 1800–1000 cm^−1^ produced PCA score plot. (**B**) Loading plot in PC-1 and 4.

The principal component analysis (PCA) is the most common multivariate analysis technique by reducing the number of variables of the data set, and then the sample will be clustered into groups depending on their characteristics, even very small variable differences from the others. Qualified FTIR spectral groups measured from the untreated HuCCA-1 cells and the cells treated with SG and cetuximab were used for identifying their biomolecular alterations. The score plot of PC-1 (57%) against PC-4 (3%) (Figure 7A) shows clusters of the three samples which are discriminated by their variables related to the loading plot (Figure 7B), especially for the asymmetric and symmetric stretching of -CH_2_- fatty lipid at 2923 and 2852, amide I at 1664, 1648, and 1617, and at 1236 cm^−1^ for the asymmetric phosphate stretching (Figure 7B).

### 2.8. The Biomolecules Alteration in HuCCA-1 Cells after Treatment with SG and Cetuximab

**Figure 8:**
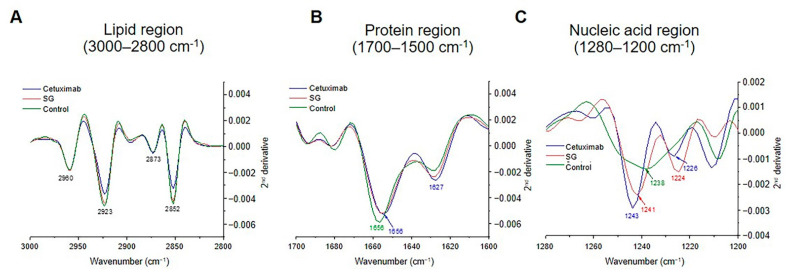
Comparison of the secondary derivative FTIR spectra of untreated HuCCA-1 cells, HuCCA-1 cells, incubated with 50 µg/mL cetuximab, and 100 µg/mL SG for 48 h. Data representing three regions: (**A**) lipid region (3000–2800 cm^−1^), (**B**) protein (1700–1500 cm^−1^), (**C**) nucleic acid region (1280–1200 cm^−1^).

To observe biomolecular alteration in the HuCCA-1 cells after treated with SG or cetuximab, averaged secondary FTIR spectra of the three samples, taken from the EMSC step, were plotted and overlaid, and loading results were used as guide direction for the interpretation. In the lipid region, two peaks at 2923 and 2852 cm^−1^ corresponded to the asymmetric and symmetric stretching of the methylene (-CH_2_) groups of membrane lipids, clearly pronounced that lipid in the cell treated by cetuximab and SG decreased comparing to control (Figure 8A). Both cetuximab and SG stimulate nucleic acid production as the peak at 1243 and 1241 cm^−1^, respectively, increased sharply, and the highest peak was shifted from 1238 to 1243 cm^−1^ (Figure 8C). Notably, the secondary structures of protein accumulation in the cells markedly changed. The α-helix was decreased while β-sheet moderately increased, and this evidence was found both in cetuximab- and SG-treated cells. This may be caused by a transition of the secondary structural protein from an α-helix to a β-sheet (Figure 8B).

## 3. Discussion

EGFR signaling has been shown to regulate different processes involved in tumor development, such as proliferation [20], migration and invasion [21]. In CCA, EGFR signaling pathways including JAK/STAT3 [22], Raf/MEK/ERK [23] and PI3K/AKT [23,24] have been shown to implicate in CCA pathogenesis. Previously, we reported the anti-proliferative effect of SG on cholangiocarcinoma cells, HuCCA-1, by arresting the cell cycle [10]. The inhibition mechanism is mediated through EGFR, mitogen-activated protein kinase (MAPK) and ERK pathway. Cetuximab is a humanized monoclonal antibody that binds to the extracellular domain of EGFR, blocks ligands, such as EGF, from binding to the receptor and activating downstream signaling [6]. Previously, phase II clinical trials showed that advanced CCA patients had low responsiveness to a single cetuximab treatment [25]. A number of reports on other cancers indicated the potential of natural product extracts to enhance the efficacy of cetuximab. For instance, germinated brown rice extract has been shown to sensitize cetuximab in colon cancers [26]. Combined treatment of curcumin and cetuximab markedly suppressed protein expression of EGFR and ERK phosphorylation in oral cancer cells [26]. Here, we have determined the effect of SG and cetuximab separately and in combination on the migration rate of CCA cells and EGFR-ERK signaling. It is well established that during cancer cell migration, up-regulation of FAK and down-regulation of E-cadherin are crucial for promoting their migration [27,28]. Our study showed that an SG and cetuximab combination exerts a synergistic anti-migration effect on CCA cells by increased suppressing of EGFR-ERK signaling. A reduced EGFR phosphorylation is connected to phosphorylated ERK1/2, which is the downstream effector of focal adhesion kinase (FAK) and E-cadherin [29,30]. Our results agree with a previous study which showed that a combination of cetuximab and brown alga polysaccharides, fucoidan had a synergistic effect on inhibiting metastasis of hepatocellular carcinoma cells by increased E-cadherin [31].

Further, we studied a possible interaction of SG with EGFR. For EGFR activation, prior to ligand binding, EGFR is in a tethered conformation in which domain II is folded into domain IV via disulfide bonds. EGFR ligands, such as EGF, are known to bind to domains I and III of EGFR monomers, which promotes a domain rearrangement and exposes the dimerization arm in domain II, leading to a stabilized extended conformation and, consequently, receptor dimerization. Here, we employed the molecular docking tool to predict a possible binding site of SG on the EGFR extracellular domain. The result showed that SG binds onto the dimerization arm (domain II) of EGFR’s extended conformation, while cetuximab, which is known to bind onto domain III and stabilize the tethered conformation, consequently prevents the receptor from adopting the untethered (extended) conformation required for dimerization [5,32]. From these findings, we proposed that SG prevents EGFR activation through its binding to the dimerization arm of the untethered conformation of EGFR on CCA cells; thereby, EGFR dimerization is destabilized, and this was validated by the dimerization assay, which showed that SG inhibited ligand-induced EGFR dimerization.

The data that SG and cetuximab bind onto different domains of EGFR probably supports the biological study that showed SG enhanced the effect of cetuximab. A possible explanation for this is that EGFR exists in a number of different conformations on the cell surface, where the tethered (closed) conformation is the most frequent. However, some of the receptors still remain in the untethered conformation, with bound ligands, and dimerize [5,32]. In this respect, cetuximab will only be effective in maintaining the tethered conformation but cannot effectively target cancer cells exhibiting other conformations [5,32,33]. We propose that SG apparently enhanced anti-cancer potency of cetuximab may be due to the dual interaction on EGFR contributes to enhanced inhibition of EGFR activation: cetuximab interferes with the tethered (closed) conformation while SG interferes with the un-tethered (extended) conformation. However, the structural mechanistic basis for inhibition of EGFR by SG requires further study.

In recent years, studies of cellular behaviors after drug treatments in a single cell have received increased attention in anti-cancer research. Synchrotron-FTIR-MS provides a biochemical spectral signature for a specific drug treatment compared to a control group [12,16,17,34]. We then employed the Synchrotron-FTIR-MS analysis to present the effects of SG on biomolecular changes of CCA cells compared with the known drug, cetuximab, for potential use as an initial screening tool. We demonstrate the FTIR spectra can discriminate between CCA cells before and after treatment with SG and cetuximab by following changes in RNA/DNA, lipids, and α-helix and β-sheet secondary protein structures from amide I vibrations. A suppression of lipid peaks in the SG- and cetuximab-treated cells suggests a decrease in cellular lipid synthesis [35], and this decreased lipid peak has been reported to be associated with apoptosis [17]. It has been shown that these changes resulted from the disturbance of membrane fluidity and translocation of phosphatidylserine during the apoptosis process [18]. Noting that cancer cells utilize lipid synthesis for new cell membranes during rapid proliferation, which provides protection from lipid peroxidation and apoptosis [36]. Overall, SG altered the protein and nucleic acids profiles of the CCA cells in a similar pattern to cetuximab, although to a greater extent. When the cells treated with SG or cetuximab were compared, it was apparent that SG initiated a greater amount of nucleic acid, induced notable changes in DNA conformation [37] and effected a decrease in protein appearing in a β-sheet form. The prominent decrease observed in the protein region was correlated with the downregulation of protein markers for EGFR signaling inside the cell. This prediction was verified by results from the in vitro study, which showed that SG and cetuximab treatments downregulated signaling proteins in EGFR/ERK pathway.

In conclusion, our study demonstrates the underlined anti-cancer effect of SG and offers a promising approach for the application of SG as a drug adjuvant that allows for a reduced effective dose of cetuximab and may enhance the drug’s efficacy for CCA patient’s treatment. Moreover, this study reveals a potential use of SR-FTIR-MS for a rapid initial anti-cancer drug screening and prediction of drugs’ effects.

## 4. Materials and Methods

### 4.1. Sulfated Galactans (SG) and Cell Lines

*G. fisheri* was extracted and purified to obtain SG as previously described [7]. SG consists of 3-linked-β-d-galactopyranose (G) and 4-linked 3,6-anhydro-α-l-galactose (LA) or α-l-galactose-6-sulfate (L6S) with partial methylation (CH3) at C-2 of LA and C-6 of G, and sulfation of C-4 and C-6 of d-galactose units (G4S and G6S) with a 90% purity by HPLC analysis (Waters, Milford, MA, USA).

Human cholangiocarcinoma cell lines established from CCA tissue of Thai patients were employed in this study. HuCCA-1 and KKU-M213 are both human intrahepatic cholangiocarcinoma cell lines derived from a patient with *O. viverrini* infection [38,39], and show overexpression of EGFR. HuCCA-1 and KKU-M213 were obtained from the Japanese Cell Research Bank (HuCCA-1 (JCRB1657) and KKU-M213 (JCRB1557)). Both cell lines were cultured in RPMI supplemented with 10% fetal bovine serum and 2 mM l-glutamine (Sigma Aldrich, St.Louis, MO, USA) in a 5% CO_2_ atmosphere at 37 °C.

### 4.2. Effect of SG and Cetuximab on Cell Viability

The effect of SG and cetuximab on CCA cell viability was examined using the methyl thiazolium bromide (MTT) assay. CCA cell lines, HuCCA-1 and KKU-M213, were grown overnight in 96-well plates to a density of 5 × 10^3^ cells/well, and cells incubated with increasing concentrations (0, 10, 50, 100 and 500 μg/mL) of SG or cetuximab for 24 h. After incubation, 100 μL of MTT solution (0.5 mg/mL) (Sigma Aldrich, St. Louis, MO, USA) was added to each well and incubated for 3 h at 37 °C in the dark, followed by 100 μL of dimethyl sulfoxide (DMSO) (Merck, Darmstadt, Germany). Cell viability was quantified by reading the absorbance at 490 nm with a Versamax microplate reader using SoftMax^®^ Pro 4.8 analysis software (Molecula Devices, Union City, CA, USA).

### 4.3. Anti-Migration Effect by Wound Scratch Assay and Analysis

HuCCA-1 cells were seeded at a density of 1 × 10^4^ cells/well and cultured overnight in 6-well plates. The culture medium was aspirated, and a sterile pipette tip used to create a scratch wound, and 5% FBS fresh medium was added before treatment with SG 50 μg/mL, cetuximab 100 μg/mL, or an SG and cetuximab combination. The cells were photographed immediately (time 0) and at 6 and 24 h after scratching using a phase-contrast microscope. The percentage of wound area was measured, using the data from time 0 (*T_0_*), the wound area (*T_t_*: 6 and 24 h) by the following formula [40]:

Percentage of wound area = 100 − (wound area at (*T_0_* − *T_t_*) / wound area at *T_0_*) × 100

### 4.4. Western Blotting

HuCCA-1 cells were grown under scratch wound healing treatment as above. Cells were collected at 12 h, and cell lysates were prepared in lysis buffer (3 mM MgCl_2_, 1 mM EGTA, 10 mM sodium pyrophosphate, 50 mM sodium fluoride (NaF) and 100X protease inhibitor solution) and centrifuged at 12,400×g for 15 min at 4 °C. The supernatant was collected to determine protein concentration by BCA assay using the PierceTM BCA Protein Assay Kit (Thermo Scientific, Waltham, MA, USA). Proteins were separated on 8% gels by SDS-PAGE, blotted onto a nitrocellulose membrane (Merck, Darmstadt, Germany), and incubated with primary antibodies: phosphorylated-EGFR (Y-1173) (p-EGFR) (1:500), EGFR (1:500), and phosphorylated-ERK1/2 (p-ERK1/2) (1:1000) (Santa Cruz Biotechnology, CA, USA), ERK (1:1000), FAK (1:1000), p-FAK (1:1000) and E-Cadherin (1:1000) (Cell Signaling Technology, Beverly, MA, USA), followed by incubation with a horseradish-peroxidase-conjugated (HRP) secondary antibody. Anti-alpha (α)-tubulin antibody (1:1000) (Santa Cruz Biotechnology, Santa Cruz, CA, USA) was also probed in all blots as an internal control. Proteins were detected using the Enhanced Chemiluminescence (ECL) kit (Bio-Rad Laboratories, Hercules, CA, USA) and visualized on Chemiluminescent gel document (Alliance Q9 mini) (UVITEC, Cambridge, UK). Protein expression was quantified by ImageJ analysis (from NIH website by Scion Corporation, Frederick, MD, USA).

### 4.5. Molecular Docking

Based on the hypothesis that the anti-cancer activity of SG with cancer cells is associated with EGFR inhibition, the docking poses for SG, cetuximab, and EGF were determined according to their interactions with the EGFR extracellular domain as previously described [41]. The binding of SG, cetuximab and EGF to EGFR was assessed using GOLD docking software version 5.6.1 (The Cambridge Crystallographic Data Centre (CCDC), Cambridge, UK) [42]. The dimer form of the extracellular domain of the EGFR model was derived from the Protein Data Bank (PDB accession code: 1MOX), and the extracellular domain was set as an input receptor. The SG model was constructed using the Discovery Studio 2018 (Accelrys Inc., San Diego, CA, USA) and was used as an input ligand. The GoldScore was used for the docking scoring. The top-ranking of docked poses from the docking result was used for further analysis. The visualized ligand docking and binding sites were analyzed by Discovery Studio 2018 (NeoTrident Technology Ltd., Beiging, China).

### 4.6. EGFR Dimerization Assay

Since EGFR dimerization is essential for activation of the EGFR pathway, we determined the effect of SG on EGFR dimerization following EGFR stimulation by the method previously described [43]. HuCCA-1 cells were pre-incubated with SG for 15 min at a final concentration of 100 μg/mL, and EGFR ligands which included murine EGF (mEGF), human EGF (hEGF), heparin-binding EGF (HB-EGF), (100 ng/mL) were added followed by the addition of bis(sulfosuccinimidyl)suberate (BS^3^; Pierce, Rockford, IL, USA), a crosslinker, at a final concentration of 3 mM for 20 min on ice. The cross-linking reactions were quenched by the addition of glycine to a final concentration of 250 mM. Cells were collected, lysed, and equal amounts of protein were loaded onto an 8% SDS-PAGE gel. After electrophoresis, proteins were transferred to the nitrocellulose membrane, and EGFR dimer was detected by Western blotting using an anti-EGFR antibody (Cell Signaling Technology, Beverly, MA, USA).

### 4.7. Synchrotron Radiation-Based Fourier-Transform Infrared Microspectroscopy (SR-FTIR-MS)

The biochemical alteration of CCA cells after treatment with SG and cetuximab was compared to untreated control cells using SR-FTIR-MS as previously described [16]. HuCCA-1 cells were grown on 22 mm-diameter × 1.0 mm thickness calcium fluoride (CaF_2_) IR transmission windows to a density of 1 × 10^4^ cells and treated with 50 μg/mL SG and 100 μg/mL cetuximab for 24 h. The floating cells or dead cells were discarded. The SR-FTIR-MS experiment was performed at the BL4.1, Synchrotron Light Research Institute (Public Organization), Nakhon Ratchasima, Thailand, using a Bruker FTIR spectrophotometer Vertex70 coupled with a Hyperion 2000 microscope with a 36 × IR objective lenses and the MCT (HgCdTe) detector (Bruker Optik GmbH, Ettlingen, Germany). OPUS 7.5 software (Bruker Optik GmbH, Ettlingen, Germany) was used as the instrumental controlling software and spectral data collection. The samples were microspectroscopically measured in the 4000–600 cm^−1^ spectral range using an aperture size of 10 × 10 µm^2^ with a 4 cm^−1^ spectral resolution. All measured spectra were individually averaged from repeated 64 -scans for obtaining high signal/noise ratios. Qualified spectra with amide I peak height between 0.3–1.2 abs in the spectral range of 3800–1000 cm^−1^ in each sample group were selected by OPUS. Savitzky-Golay smoothing using a third polynomial order and nine smoothing points together with linear baseline correction and EMSC were pre-set prior to PCA analysis by using the Unscrambler^®^ X 10.5 software (CAMO Software AS, Oslo, Norway) as described previously [44].

### 4.8. Statistical Analysis

All methods were performed in triplicate. Data are presented as means ± SEM and statistically analyzed by one-way analysis of variance (ANOVA) followed by Turkey’s multiple comparison tests, and within-treatment group comparisons were performed using paired *t*-test in GraphPad Prism program version 6.0 (GraphPad Software Inc., San Diego, CA, USA). A difference with a *p-*value less than 0.05 was considered statistically significant.

## Figures and Tables

**Table 1:** SR-FTIR-MS band assignments for functional groups found in the second derivative spectra of HuCCA-1 cells.

2nd Derivative Peak (cm^−1^)	Band Assignments
2960	-CH_3_ and -CH_2_- asymmetric stretching
2923	-CH_3_ and -CH_2_- asymmetric stretching
2873	-CH_3_ and -CH_2_- symmetric stretching
2852	-CH_3_ and -CH_2_- symmetric stretching
1656	Alpha helix of amide I
1627	Beta sheet of amide I
1224, 1226, 1238, 1241, 1243	P=O phosphodiester bond from nucleic acid

## Data Availability

Not applicable.
